# Image-guided locoregional non-intravascular interventional treatments for hepatocellular carcinoma: Current status

**DOI:** 10.1016/j.jimed.2020.10.008

**Published:** 2020-10-12

**Authors:** Kun Qian, Feng Zhang, Stephen K. Allison, Chuansheng Zheng, Xiaoming Yang

**Affiliations:** a Department of Radiology, Union Hospital, Tongji Medical College, Huazhong University of Science and Technology, Wuhan, Hubei, 430022, China; b Department of Radiology, Image-Guided Biomolecular Intervention Research, University of Washington School of Medicine, Seattle, WA, 98109, USA; c Division of Interventional Radiology, Department of Radiology, University of Washington School of Medicine, Seattle, WA, 98109, USA

## Abstract

Hepatocellular carcinoma (HCC) is one of the most deadly and frequent cancers worldwide, although great advancement in the treatment of this malignancy have been made within the past few decades. It continues to be a major health issue due to an increasing incidence and a poor prognosis. The majority of patients have their HCC diagnosed at an intermediate or advanced stage in theUSA or China. Curative therapy such as surgical resection or liver transplantation is not considered anoption of treatment at these stages.

Transarterial chemoembolization (TACE), the most widely used locoregional therapeutic approach, used to be the mainstay of treatment for cases with unresectable cancer entities. However, for those patients with hypovascular tumors or impaired liver function reserve, TACE is a suboptimal treatment option. For example, embolization does not result in complete coverage of a hypovascular tumor, and may rather promotes postoperative tumor recurrence, or leave residual tumor, in these TACE-resistance patients. In addition, TACE carries a higher risk of hepatic decompensation in patients with poor liver function or reserve.

Non-vascular interventional locoregional therapies for HCC include radiofrequency ablation (RFA), microwave ablation (MWA), high-intensity focused ultrasound (HIFU), laser-induced thermotherapy (LITT), cryosurgical ablation (CSA), irreversible Electroporation (IRE), percutaneous ethanol injection (PEI), and brachytherapy. Recent advancements in these techniques have significantly improved the treatment efficacy of HCC and expanded the population of patients who qualify for treatment. This review embraces the current status of imaging-guided locoregional non-intravascular interventional treatments for HCCs, with a primary focus on the clinical evaluation and assessment of the efficacy of combined therapies using these interventional techniques.

## Introduction

Hepatocellular carcinoma (HCC) is a major cause of global cancer-related deaths, and its incidence and mortality rates are increasing worldwide.^1^ Despite improvements in surveillance programs and diagnostic techniques for HCC, most patients are diagnosed at the intermediate-to-advanced stage, and only a small percentage of newly diagnosed patients are eligible for curative treatments such as ablation, surgical resection, or liver transplantation.^2^ Over the past few decades, catheter-based imaging-guided embolic therapies, such as transarterial chemoembolization (TACE), have become representative of the standard interventional treatment for patients with intermediate-to-advanced-stage HCCs. TACE involves delivery of chemotherapeutic drugs and embolization agents into tumor-feeding arteries to target tumor cells while sparing the normal hepatic parenchyma. The Barcelona-Clinic Liver Cancer Group recommends TACE as the first line of therapy for patients with tumors at the intermediate stage; the effects of TACE have been corroborated by robust evidence.^3^ However, the treatment effect varies due to several factors, including lack of standardization of treatment methodology, unanimous patient selection criteria, and/or the type of regimen used. Moreover, treatment with TACE does not attain ideal long-term survival rates in patients, mainly because only partial necrosis of the tumor is achieved in a relatively large number of patients, and the rates of postoperative tumor recurrence are high.^4^ Thus, TACE cannot be exclusively used for the treatment of all HCC patients.

In recent years, advances in interventional radiological techniques have enabled the development of alternative image-guided locoregional interventional modalities that play important roles in the treatment of HCC. Imaging-guided radiofrequency ablation (RFA) is currently used as an alternative to surgery in selected patients. It is considered a safe and effective treatment for patients with early-to-intermediate HCC when surgical resection is not feasible.^5^ Compared with RFA, microwave ablation (MWA) can obtain a broader zone of active heating and produce higher temperatures within the targeted area in a shorter amount of time, thereby reducing back-heating effects.^6^ Other locoregional therapies, such as high-intensity focused ultrasound (HIFU), laser-induced thermotherapy (LITT), cryoablation, irreversible electroporation (IRE), percutaneous ethanol injection (PEI), and brachytherapy, also show promising results in the treatment of HCC.^2^ The wide range of available treatment options implies that clinicians must determine the optimal interventional therapy on the basis of their knowledge and experience. Further, stringent clinical trials are required to evaluate the therapeutic efficacy of these techniques. In this review, we assess the current status of imaging-guided locoregional non-vascular interventional ablation treatments for HCC, primarily focusing on clinical evaluation and assessment of the efficacy of combined therapies.

## Ethical approval

The study was approved by the ethics committee of the University of Washington. All clinical practices and observations were conducted in accordance with the Declaration of Helsinki. Informed consent was obtained from each patient before the study was conducted.

## Thermal ablation

Imaging-guided thermal ablation of tumors is performed by local application of thermal energy at temperatures exceeding 50 ​°C to tumor tissues to induce irreversible cell injury, tumor apoptosis, and copulative necrosis.^7^ Currently, thermal ablation techniques used for locoregional treatment of HCCs include RFA, MWA, LITT, HIFU, and IRE.

### Radiofrequency ablation

Following the first application of RFA to hepatic tumors in the early 1990s, this technique has been increasingly used for the treatment of hepatic tumors (Table 1). To achieve RFA-induced tumor tissue death, radiofrequency (RF) electrodes are used to induce a high-frequency alternating current, which causes high-frequency friction of water molecules in tumor tissues and generates high temperatures locally. This effect leads to copulative necrosis and protein denaturation inside the tumor tissue. RFA is a safe procedure; it has a very low mortality rate of 0.15% and leads to treatment-related severe adverse events in only 4.1% of cases.^8^ Being a less invasive procedure than surgery, the importance of RFA has been well-demonstrated in multiple study series, with 1-, 3-, and 5-year disease-free survival (DFS) rates of 30–70%, 20–49% and 10–40%, respectively, and 1-, 3-, and 5-year overall survival (OS) rates of 75–99%, 40–90%, and 30–75%, respectively. One study compared 639 HCC patients treated with redo hepatic resection (RHR) and RFA and found no differences in the 1-, 3-, and 5-year DFS or OS rates between the two groups.^9^

**Table 1:** Results of RFA for HCCs.

Article	Region	Patient NO.	Tumor diameter	Overall survival rate (%)		
				1-year	3-year	5-year
Zhang, L. et al. (2015).	China	837 RFA	≤3 ​cm	97.0	71.0	54.0
Parisi, A. et al. (2013).	Italy	87 HR vs	≤6 ​cm	89.7	72.4	40.2
		53 RFA		93	43.4	22.6
Tohme, S. et al. (2013).	USA	50 HR vs	≤3 ​cm	88.0	68.0	47.0
		60 RFA		86.0	50.0	35.0
Hyun, D. et al. (2017).	Korea	69 TACE ​+ ​RFA	≤3 ​cm	100.0	89.0	80.0
Liu, H. et al. (2016).	China	100 TACE ​+ ​RFA	Median 2.8 ​cm	96.0	67.2	45.7
Song, K. D. et al. (2016).	Korea	61 TACE ​+ ​RFA	≤3 ​cm	100.0	91.9	83.6
Tang, Z. et al. (2017).	China	60 Sorafenib ​+ ​RFA	≤3 ​cm	91.1	72.8	57.5
Chen, K. et al. (2014).	China	68 Iodine-125 + RFA	≤3 ​cm	100.0	86.7	66.1

HR: Hepatic resection; RFA: Radiofrequency ablation; TACE: Transarterial chemoembolization.

The patient selection process for RFA is generally based on the Barcelona Clinic Liver Cancer (BCLC) staging system. RFA treatment is indicated for patients with very early (Stage 0) to early stage (Stage A) cancer, Eastern Cooperative Oncology Group (ECOG) performance status of 0, Child-Pugh class A or B liver profile, and three or fewer HCC nodules ​< ​3 ​cm in size. In one study, researchers treated 604 patients with either hepatic resection or RFA for a single HCC ≤3 ​cm in size. They showed that RFA is an effective alternative, with an OS comparable to that of hepatic resection for such single small HCCs.^10^ Another study included 91 patients who were initially diagnosed as having intermediate-stage HCC and were treated with RFA. The results showed that RFA was useful for the treatment of less advanced intermediate-stage HCC and could be used as an alternative to TACE in select cases.^11^ One group of researchers used RFA to treat patients with HCC lesions >3 ​cm, staged A–B2 as per the BCLC criteria, with multiple electrode needles. They were able to demonstrate that RFA is a useful and safe therapeutic approach.^12^ On combining RFA with TACE, Saviano et al. revealed that RFA is effective in patients with a solitary HCC ≥3 ​cm.^13^ Several factors such as tumor size, number, location, liver function, comorbidity, and initial response contribute to OS, indicating that intermediate-to-advanced-stage HCCs can be considered indications for RFA when patients are properly evaluated and selected. In addition, RFA can serve as a first-line stand-alone bridge therapy for liver transplantation with excellent long-term OS, as it has a low dropout rate among patients with tumor progression, despite the long waitlist for liver transplantations.^14^ RFA can also be performed in combination with other therapies, including percutaneous ethanol injection (PEI), sorafenib therapy, iodine-125 implantation, and hepatic resection via a laparoscopic or laparotomic approach.^15^, ^16^, ^17^, ^18^, ^19^, ^20^, ^21^ In addition, new techniques are being developed to increase the area of ablation necrosis induced by RFA, such as improvement of electrode needles; hydrochloric acid or hypertonic saline injections during RFA; and combination with immunotherapy, gene therapy, and novel antitumor drugs.^5^^,^^22^, ^23^, ^24^, ^25^ Although RFA is currently the most widely used modality of the various thermal procedures, large-scale clinical trials are required to confirm the effectiveness of these combined therapies.

### Microwave ablation

In the early 1980s, MWA was used during surgery for hemostasis and excising tissues for locoregional treatment of hepatic tumors. MWA creates an electromagnetic field in the tissue, causing high-frequency rotation of water molecules, which induces thermal injury to the tumor tissue, leading to coagulation necrosis.^26^ Microwave-induced thermal energy can create larger areas of ablation in a shorter amount of time than RFA. Hence, MWA is considered more suitable for the treatment of larger and highly perfused tumors close to sizable vessels, a condition wherein the effect of RFA is compromised because of heat-sink effects (HSE) from blood flow in large vessels. Regarding safety and efficacy, a meta-analysis has shown no significant differences between MWA and RFA in terms of 1- and 5-year OS, DFS, complete ablation (CA), and localized recurrence rate (LRR).^27^ (Table 2).

**Table 2:** Results of MWA for HCCs.

Article	Region	Patient NO.	Tumor diameter	Overall survival rate (%)		
				1-year	3-year	5-year
Xu, Y. et al. (2017).	China	142 MWA	1.0–5.0 ​cm	97.2	75.4	50.6
Thamtorawat, S. et al. (2016).	USA	173 MWA	≤3.0 ​cm (118)	91.2	82.1	–
			3.1–5.0 ​cm (55)	92.3	83.9	–
Ding, J. et al. (2017).	China	132 MWA exophytic (71)	≤3 ​cm	100	75.7	52.9
		non-exophytic (61)		95.0	73.8	61.5
Xu, Y. et al. (2016).	China	82 MWA	5.0–6.0 ​cm	92.7	63.4	41.1
Shi, J. et al. (2014).	China	117 MWA vs 107 HR	3.0–5.0 ​cm	94.0	70.0	52.0
				94.0	72.0	60.0
Ma, S. et al. (2017).	China	433 MWA	≤10 ​cm	83.5	58.7	–
Huang, S. et al. (2014).	China	452 MWA	1–7 ​cm	93.0	79.0	57.0
Ni, J. Y. et al. (2014).	China	86 TACE ​+ ​MWA	3–10 ​cm	72.1	31.4	13.9

HR: Hepatic resection; MWA: Microwave ablation; TACE: Transarterial chemoembolization.

There is substantial evidence to indicate that MWA is equivalent to RFA for the treatment of early-stage HCC.^28^ In recent years, numerous types of MWA electrodes and generators have been developed to increase the size of ablation zones and achieve larger ablation margins, with the ultimate goal of decreasing local tumor progression. MWA can be performed using a computed tomography (CT)-guided stereotactic navigation system, ensuring high efficacy and safety for HCCs in challenging locations such as the hepatic dome and subcapsule, with tumor sizes of up to 5 ​cm in diameter.^29^, ^30^, ^31^, ^32^ It has been reported that MWA results in similar local responses and long-term outcomes for HCC patients with exophytic or non-exophytic subcapsular tumors.^33^^,^^34^ Some researchers have successfully combined MWA with laparoscopic splenectomy (Lap-Sp) for the treatment of HCC with cirrhotic portal hypersplenism, suggesting that simultaneous MWA and Lap-Sp can achieve safe and effective treatment of HCC in this subgroup.^35^ Another study found that MWA, when combined with TACE, is superior to RFA with TACE for treating unresectable single masses ​> ​5 ​cm in size, with more effectiveness in terms of tumor response.^36^ In that study, 66 patients with 72 large HCC lesions (≥5 ​cm; average size, 9 ​cm ​± ​3.9 ​cm) were treated with ultrasound (US) imaging-guided MWA, immediately followed by TACE. This combination therapy achieved a median progression-free survival (PFS) of 9 months and 6-, 12-, and 18-month OS rates of 93.9%, 85.3%, and 66.6%, respectively.^37^ However, there are some controversies regarding this technique. One study evaluated the safety and efficacy of MWA in treating single-nodule focal hepatic malignancies and found that tumor size did not appear to influence complete ablation rates or local recurrence.^38^ Another study demonstrated that when using MWA for the treatment of HCC, local tumor progression was significantly correlated with the hepatic arteries within the ablation zone but not with hepatic or portal veins.^39^ Further, a series of randomized studies with large sample sizes are necessary to evaluate the efficacy of MWA in treating HCCs sized 3–5 ​cm.

### High-intensity focused ultrasound

HIFU, a non-invasive image-guided thermal ablation technique, uses acoustic lenses or curved piezoelectric transducers to deliver high-energy US waves designed to pass through normal tissues and target specific spots of tumor tissues.^40^ When compared with medical diagnostic US imaging, HIFU uses lower frequencies with significantly higher energies. HIFU treatment is often guided by magnetic resonance imaging (MRI), known as magnetic resonance (MR)-guided focused US.

In the past few years, HIFU has been explored as a treatment for unresectable, advanced-stage HCC and liver metastases. Thus far, studies have shown that HIFU is a safe and well-tolerated therapeutic modality that is able to induce copulative necrosis in select tumor tissues without damaging adjacent normal structures; it has even been used in Child-Pugh C patients.^41^^,^^42^ A previous study did not find any difference in terms of the 1- and 3-year OS rates between HIFU and RFA in patients with HCCs smaller than 3 ​cm (97.4% vs. 94.6% and 81.2% vs. 79.8%, respectively, P ​= ​0.530).^43^ A similar study compared the outcomes of HIFU ablation with those of TACE and concluded that the OS rates of the HIFU group (1-, 3-, and 5-year OS rates of 84.6%, 49.2%, and 32.3%, respectively) were significantly higher than those of the TACE-only group (1-, 3-, and 5-year OS rates of 69.2%, 29.8%, and 2.3%, respectively) (P ​= ​0.001).^44^ Researchers have demonstrated that HIFU ablation is safe and effective as a bridging therapy for patients with HCC who are waiting for organ transplantation.^45^^,^^46^ New therapeutic agents, such as microtubules and nanoparticles that function as synergistic agents to enhance HIFU efficacy and prevent tumor recurrence due to heterogeneous ablation, are currently being developed to improve the curative effects of HIFU.^47^, ^48^, ^49^

In certain cases, HIFU ablation needs to be performed in centers with highly experienced practitioners. When compared with the numerous clinical studies on MWA and RFA, the studies on HIFU have been limited. Studies with large cohorts are required to investigate the long-term efficacy of HIFU in treating HCC.

### Laser-induced thermotherapy

Interstitial LITT was first introduced in the mid-1990s as an alternate imaging-guided thermal ablation technique to treat liver tumors, after an earlier study showed that LITT yielded remarkable local tumor control.^50^ LITT is performed by inserting an optical fiber into the tumor tissue to deliver high-energy laser radiation with a wavelength of 1046 ​nm, thereby inducing tumor destruction. LITT generates substantial thermocoagulation in tumor tissues via the absorption of laser light with temperatures ranging from 45 to 55 ​°C, or by short-term exposure to laser radiation at temperatures higher than 60 ​°C.

LITT was traditionally performed under the guidance of CT and/or US imaging. Later, MRI was developed to guide placement of the laser applicator and MR thermometry was used to simultaneously monitor thermocoagulation.^51^ Thus, MRI with specific sequences can not only guide placement of laser probes but also allow accurate real-time evaluation of the extent of thermal ablation. In one study, 113 HCC patients were treated with MRI-guided LITT, and the therapeutic potential of this technique was evaluated. The median survival period for all patients was 3.5 years, and the 1-, 2-, 3-, and 5-year OS rates were 95%, 72%, 54%, and 30%, respectively. The same group of researchers also demonstrated the therapeutic effect of LITT on up to five liver metastatic lesions <5 ​cm in diameter.^52^, ^53^, ^54^ In one study, LITT was combined with TACE to treat patients with unresectable liver metastases. The findings revealed that tumor size could be reduced in 50.6% of the cases with repeated TACE.^55^ Alongside neo-adjuvant treatment of TACE, MR-guided LITT can be used to treat large-sized (5–8 ​cm) HCCs.^56^ Another study compared LITT with RFA and found that the mean OS was 42 months for both groups, with 1- and 3-year survival probabilities of 94% and 80%, respectively, for the laser ablation group and 94% and 89%, respectively, for the RFA group.^57^ A separate study also confirmed that LITT offers an invaluable alternative for thermal ablation of small HCCs in cirrhotic patients.^58^

Imaging-guided LITT is safe and has a major complication rate of 1.5% and a minor complication rate of 6.2%. Major complications are associated with excessive energy deposition in high-risk nodule locations, whereas minor complications are associated with excessive energy, high bilirubin levels, and low prothrombin time when performing LITT.^59^ Patients have a good tolerance for image-guided LITT with a low degree of side effects.^60^ As with other thermal ablations, further prospective and comparative studies are required to establish the role of LITT in the treatment of HCCs.

### Cryoablation

Unlike heat-based ablations, cryoablation is the process of cyclic application of extremely low temperatures (−20 ​°C to −40 ​°C) to tumor tissue, and it is based on the Joule–Thomson effect. A temperature conductive applicator is inserted into the tumor, resulting in the formation of intracellular and extracellular ice crystals, thereby leading to cell death. A slow rate of freezing favors the formation of extracellular ice crystals, leading to a change in osmolality within the extracellular space that in turn induces cell dehydration and death.^61^

Cryoablation has been used to treat a variety of tumors, such as renal cell carcinoma,^62^ pancreatic cancer and prostate cancer,^63^^,^^64^ and it is used as a potentially curative therapy for inoperative HCC.^65^ Imaging-guided percutaneous cryoablation is a safe procedure for the treatment of subcapsular high-risk HCCs adjacent to major organs such as the gall bladder, colon, stomach, kidneys, diaphragm, or abdominal wall.^66^ Several studies have confirmed that cryoablation is a safe and effective procedure for patients with HCC, with 1-, 3-, and 5-year cumulative OS rates of 98.6%, 80.6%, and 60.3%, respectively (Table 3).^67^, ^68^, ^69^ Cryoablation-related minor complications have been observed in 10–20% of patients, but major complications are rare.

**Table 3:** Results of cryoablation for HCC.

Article	Region	Patient NO.	Tumor diameter	Overall survival rate (%)		
				1-year	3-year	5-year
Yang, Y. et al. (2012).	China	300 cryoablation	1.9–15 ​cm	80	32	–
Rong, G. et al. (2015).	China	866 cryoablation	≤5 ​cm	98.6	80.6	60.3
Li, Z. et al. (2013).	China	58 surgery vs	≤3 ​cm	100	77.59	70.69
		24 cryoablation		100	75	66.67
Wang, C. et al. (2015).	China	180 RFA vs 180 cryoabation	≤4 ​cm	97	67	40
				97	66	38
Huang, C. et al. (2016).	China	60 TACE ​+ ​cryoablation	Median 5.28 ​cm	54.3	22.6	6.5

RFA: Radiofrequency ablation; TACE: Transarterial chemoembolization.

Cryoablation has been demonstrated to be a relatively painless procedure when compared with heat-based ablation. Cryoablation offers several potential advantages over RFA, especially in terms of creation of larger ablation zones using multiple cryoprobes, which can simultaneously generate large ice balls within the targets. During ablation, the ice ball can be readily visualized using intraprocedural CT, MRI, or US.^70^ One study showed a significantly lower local tumor progression rate in the cryoablation group than in the RFA group (7.7% vs. 18.2%, P ​= ​0.041) for patients with HCC >3 ​cm in diameter; however, cryoablation and RFA can both achieve similar 5-year survival rates.^71^ When cryoablation was combined with TACE, the survival rate was significantly higher than that with TACE alone. Several factors, such as patient age, tumor diameter, tumor periportal location, and liver function reserve can significantly influence OS.^72^ The disadvantages of cryoablation include reperfusion in the ablation zone during melting of the ice ball, leading to a rapid release of cellular debris into systemic circulation. This surge of cellular debris in the systemic circulation may explain the higher rate of systemic complications such as cryo-shock observed after cryoablation.^73^

## Irreversible electroporation

IRE ablation is a tumor ablation technique that applies electric field pulses to cells, causing alteration or destruction of cell membranes. In contrast to reversible electroporation (RE), permanent permeability of the cell membrane induced by IRE disrupts cell homeostasis, resulting in cell death. This mechanism was validated using mathematical analysis, which predicted that IRE can be used to ablate substantial volumes of tissue without any detrimental thermal effects. IRE preserves surrounding healthy tissue and vital structures such as blood vessels, nerves, bile duct, and neighboring organs. This advantage implies that IRE can potentially be used for the treatment of HCC involving blood vessels or bile ducts.

In contrast to thermal ablation, IRE ablation is a non-thermal ablation technique without the complications of HSE or unwanted tissue damage. The procedure of IRE for HCC can be guided by CT or US, and the ablated zone can be assessed using MRI, CT, and contrast-enhanced US (CEUS).^74^ Early results are encouraging and suggest equivalence to the outcomes obtained from thermal ablation for appropriately selected, small (<3 ​cm) tumors.^75^^,^^76^ A retrospective analysis evaluated survival times after using IRE to treat tumors that are neither resectable nor eligible for thermal ablation. They found an average survival time of 24.5 months for tumor diameters ≤3 ​cm in size and 12.9 months for tumors >3 ​cm in size.^77^ IRE is expected to achieve complete ablation in patients with HCC who have had contraindications to other ablative techniques.^78^ However, there are no reports on the long-term efficacy of IRE ablation for the treatment of HCC.

## Chemical ablation

During chemical ablation, injected sclerosants diffuse into the cell and causes immediate dehydration of the cytoplasm and subsequent necrosis. Chemical ablation is commonly performed by injecting either acetic acid or ethanol into the tumor, which are suitable for HCC-endemic regions facing limited medical resources or unavailability of other tumor ablation techniques.^79^ Chemical ablation is a safe, technically simple, and an effective method for treating HCC.^80^

### Percutaneous ethanol injection (PEI)

PEI is a well-established chemical ablation technique for the treatment of small HCCs. The injected absolute ethanol can induce cellular dehydration, protein denaturation, and chemical occlusion of small tumor vessels. PEI is indicated for patients with small HCCs, and its effectiveness in treating HCCs <1.5 ​cm in size in terms of OS is equivalent to that of RFA.^81^ The major limitation of PEI is high local recurrence when treating tumors >3 ​cm in size.^82^ PEI is unable to create a safety margin for ablation, and thus does not destroy satellite lesions even in small tumors. Recent studies have shown that the combination of PEI with TACE or RFA in the treatment of small HCCs provides comparable survival rates and recurrence-free survivals.^16^^,^^83^, ^84^, ^85^ Moreover, other studies report that there are certain circumstances under which PEI therapy is a better strategy for controlling liver tumors than is RFA, especially for liver tumors located within 10 ​mm of the capsule, close to vital organs and the diaphragm, or in contact with a vessel >3 ​mm in diameter.^86^^,^^87^ PEI is believed to be effective for treating small HCCs and hypovascular tumors for which chemoembolization alone is less productive. Using PEI to treat tumors >5 ​cm in size is particularly challenging, even when combined with other locoregional therapies (Table 4). Although PEI has the primary advantage of low cost, it has been increasingly supplanted by other ablation technologies.

**Table 4:** Results of PEI for HCC.

Article	Region	Patient NO.	Tumor diameter	Overall survival rate (%)		
				1-year	3-year	5-year
Silva, M. F. et al. (2014).	Brazil	79 PEI	0.9–5 ​cm	79	48	37
Shi, F. et al. (2016).	China	180 PEI ​+ ​RFA	≤4 ​cm	87.9	57.6	38.4
Pompili, M. et al. (2015).	Italy	136 RFA vs	≤2 ​cm	97.7	77.1	62.3
		108 PEI		97	83.3	64.6
Yu, S. J. et al. (2016).	China	288 RFA vs	≤1.5 ​cm	94	81	60
		247 PEI		95	79	61
Chen, S. et al. (2017).	China	57 PEI ​+ ​RFA	≤5 ​cm	69.5	37.8	33.1
Koda, M. et al. (2001).	Japan	26 PEI ​+ ​TACE	≤3 ​cm	100	80.8	40.4

PEI: Percutaneous ethanol injection; TACE: Transarterial chemoembolization; RFA: Radiofrequency ablation.

## Technological advances from science to practice

Among the different interventional oncologic techniques, thermal ablations (primarily RFA and MWA) have been recognized as principal tools in the treatment of unresectable primary and metastatic solid malignancies. They can be used for up to 3–5 lesions in a single treatment session.^12^ However, these techniques are currently best suited only for the treatment of small lesions (<3 ​cm).^88^ When faced with medium-sized (3–5 ​cm) and particularly large (5–7 ​cm) tumors, these ablation techniques are usually limited by incomplete ablation at the tumor periphery, leaving a positive tumor margin. This incomplete tumor ablation has been attributed to several factors, including: 1) RFA heat diverted by neighboring vasculatures (HSE); 2) intentional avoidance of MWA overheating by the operator in order to protect adjacent normal structures, because of low predictability of ablation zones and rapid rise to peak temperatures within short time periods; 3) infiltrative growth patterns, micro-satellite lesions, or micro-venous tumor emboli at the 1 ​cm surgical margin of ablated tumors; 4) off-center positioning of the RFA electrode due to limited access windows.^89^^,^^90^ Ultimately, residual viable tumor cells at the tumor periphery serve as the main source of tumor persistence and recurrence, leading to treatment failure.

Recent studies from different groups have successfully confirmed that image-guided interventional radiofrequency hyperthermia (RFH), at a sub-lethal temperature ​< ​60 ​°C, can greatly enhance chemo-, gene, and immuno-therapies for malignancies in various organs.^91^, ^92^, ^93^, ^94^, ^95^ The mechanism of RFH-enhanced therapy, as currently understood, includes tissue fracture via heating, increased permeability of cytoplasmic membranes, disruption of cellular metabolism, deactivation of membrane associated pumps, and activation of the heat shock protein pathway.^7^^,^^96^ These mechanisms effectively facilitate the entry of therapeutic agents into target tumor cells, thereby promoting destruction of tumor tissue.

**Fig. 1:**
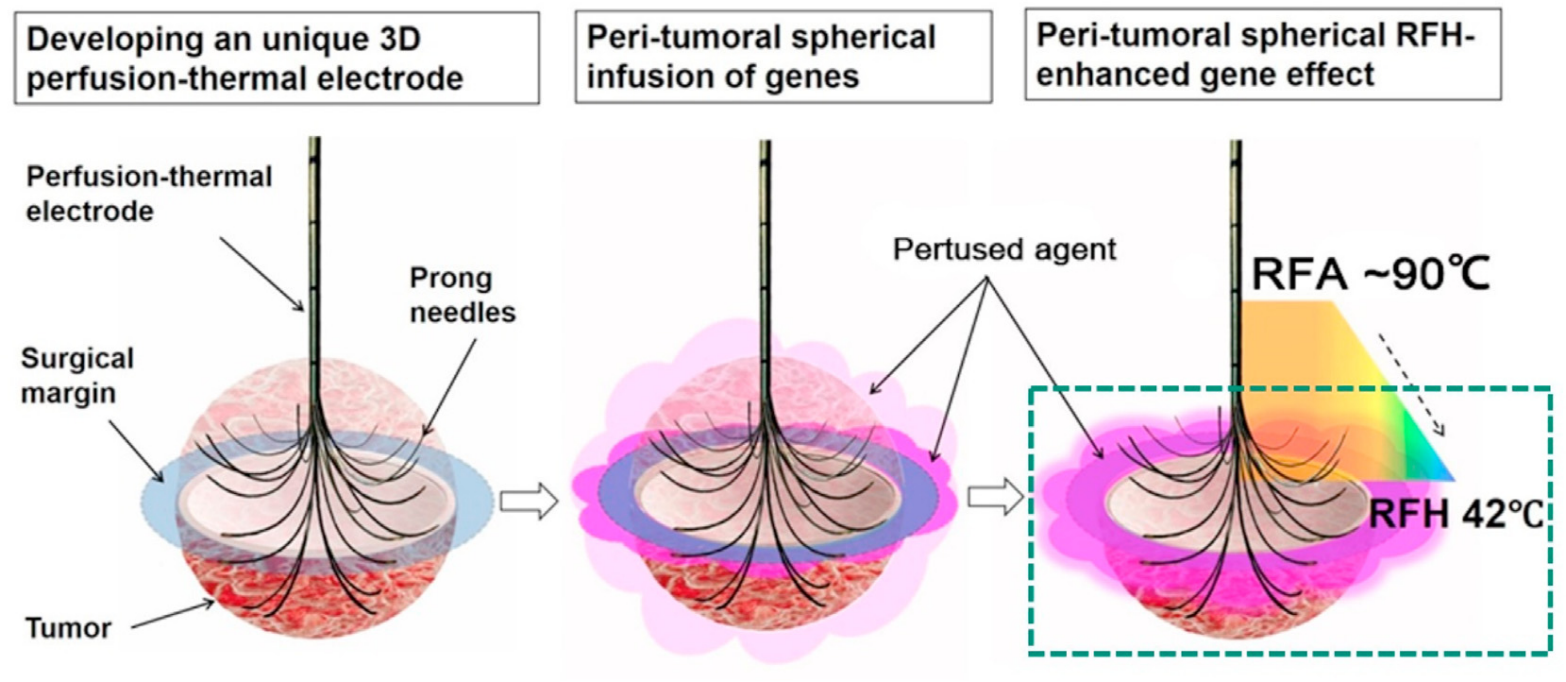
Development of a 3D perfusion-thermal electrode permits (i) creation of a central RFA lethal heat (>60 ​°C) for necrotic ablation at the tumor center; and (ii) direct spherical infusion of therapeutics into the tumor periphery. The temperature gradient from the central lethal ablation heat (>60 ​°C) results in a spherical, peri-tumoral sublethal hyperthermia (RFH, <60 ​°C), which increases therapeutic entry to the tumor cells, thereby further enhancing destruction of tumor margins.

To address the critical clinical problem of residual and recurrent tumors following RFA of medium-to-large-sized malignancies, scientists have developed new techniques to permit: 1) creation of RFA lethal heat (60–100 ​°C) for necrotic ablation at the tumor center; 2) direct spherical infusion of therapeutic agents into the tumor periphery (surgical margin). The temperature gradient from central RFA lethal heat results in a peri-tumoral sublethal RFH (42 ​°C), which increases therapeutic uptake and cytotoxicity and further enhances destruction of tumor margins (Fig. 1). This innovative combination achieves complete eradication of all viable tumor cells in targeted lesions while salvaging surrounding normal structures. This concept may open new avenues for the effective treatment of medium-to-large-sized malignant tumors of not only the liver but other solid organs as well.

## Conclusion

HCC is still a major public health problem with high global mortality rates. Image-guided thermal ablation technology, primarily RFA and MWA, plays a principal role in the treatment of non-surgical candidates with small-sized (<3 ​cm) HCCs, whereas the combination of thermal ablations with other therapies provides synergistic treatments for medium- (3–5 ​cm) and large-sized HCCs. Recent advances in brachytherapy, primarily transarterial radioembolization (TARE), aid in treating patients with portal vein thrombosis. Other ablation techniques, such as HIFU, cryoablation, and IRE, also offer viable complements to thermal ablation. Continuing scientific efforts have focused on eradicating medium-to-large-sized HCCs, for which synergistic treatment with combination therapies is currently the best option.

## Patient consent

Witten informed consent was obtained from patients for publication of these case reports and any accompanying images.

## Supported by

This work was supported by grants from the 10.13039/501100001809National Natural Sciences Foundation of China (NO. 81701800) and the Joint Funds of Union Hospital, 10.13039/501100003397Huazhong University of Science and Technology, Republic of China (NO.000003720) and the Key Laboratory of Molecular Imaging of Hubei Province, China (NO. 000003962), the key program of 10.13039/501100001809national natural science foundation of China (no. 81430040), and the 10.13039/100000002National Institutes of Health grant (R01EB012467).

## Declaration of competing interest

The authors declare that they have no known competing financial interests or personal relationships that could have appeared to influence the work reported in this paper.
